# A Case of Atypical Delayed and Prolonged Hematologic Toxicity With Azacitidine in Chronic Myelomonocytic Leukemia (CMML) and Review of Literature

**DOI:** 10.4084/MJHID.2012.017

**Published:** 2012-03-13

**Authors:** Elena Elli, Caterina Cecchetti, Angelo Belotti, Lorenza Borin, Enrico Maria Pogliani

**Affiliations:** Divisione di Ematologia, Ospedale San Gerardo – Università degli studi Milano Bicocca, Monza, Italia

## Abstract

Hypomethylating drugs are useful and have been approved for the treatment of myelodysplastic syndromes (MDS) and Chronic Myelomonocytic Leukemia (CMML). However, phase 2 and 3 studies that assessed these agents in MDS, have included only a small number of patients with CMML, and there are just a few specific reports on CMML patients. The Azacitidine is actually authorised for the treatment of CMML patients with 10–29% marrow blasts without myeloproliferative disorder, who are not eligible for haematopoietic stem cell transplantation. This hypomethylating agent in MDS is known for causing transient cytopenias, most often occurring during the first 2 cycles. Here we report a case of an atypical delayed and prolonged hematologic toxicity during Azacitidine treatment in a CMML patient; furthermore we also reviewed the literature regarding the efficacy of the drug and the management of hematologic adverse effects, in term of dose adjustments or alternative schedule of administration, in specific CMML setting.

## Introduction

CMML is a clonal disorder of haematopoietic stem cell, often occurring in elderly patients, sharing heterogeneous clinical and morphological features of both MDS and chronic myeloproliferative disorders (MPD). Originally it was classified by the French-American-British (FAB) working group as a myelodysplastic syndrome characterized by monocytosis (> 1000/microl), bone marrow (BM) monocyte infiltration, blast cells less than 5% in the peripheral blood (PB) and less than 30% in the BM.^1^ CMML has been reclassified and it is currently included in the Myelodysplastic/Myeloproliferative Neoplasms (MDS/MPN) category in the new WHO classification. Particularly, this classification subdivides CMML in two subclasses according to the prognosis: CMML-1 with 5–9% BM blasts and <5% PB blasts and CMML-2 with 10–19% BM blasts and/or 5–19% PB blasts.^2^ A WBC of 13 × 10^9^/l or greater identifies myeloproliferative variant of CMML (MPD-CMML), whereas a lower white blood cell count (WBC) defines a myelodysplastic variant of CMML (MDS-CMML).^3^

CMML is a very hard disease to treat and the prognosis of the elderly is quite variable, with an approximately 2.5-year median survival.^4^ Advanced age and comorbidities impact on survival, making bone marrow transplantation seldom possible. Patients are usually treated with supportive care or mild cytoreductive therapy (Etoposide, Hydroxyurea or low-dose Cytarabine), until leukemic evolution.^1^ In the era of epigenetics therapy, new molecular targeted therapies offer the chance to modify the natural history of this disease. Among these, the hypomethylating agents Azacitidine^5^ and Decitabine^6^ have been shown to induce responses in CMML. Pooled Azacitidine and Decitabine published studies in MDS suggest an overall response rate of 39% to 45% and an overall survival (OS) benefit for responders. However, CMML patients included in these series, represented a minority of the population.^7^,^8^

Since 2008 Azacitidine has been authorised in the European Union for the treatment of CMML patients with 10–29% marrow blasts, without myeloproliferative disorder, who are not eligible for haematopoietic stem cell transplantation.^9^–^10^

Azacitidine in MDS is known for causing cytopenias, most often occurring in the first 2 cycles. Generally, these hematologic adverse events decrease during subsequent cycles and are usually managed with dosing delays (23–29%).^11^ However, the activity of Azacitidine has been investigated in trials primarily designed to test the efficacy and safety of the drug for the treatment of MDS.^7^ Only a few reports analyzed the use of Azacitidine in specific CMML subset and there are no prospective studies with a sufficient number of patients affected by this disease.^5^,^8^

Here we report a case of an atypical delayed and prolonged hematologic toxicity during Azacitidine treatment in a patient affected by CMML; furthermore we also reviewed the literature regarding the efficacy of the drug and the management of hematologic adverse effects, in term of dose adjustments or alternative schedule of administration, in specific CMML setting.

## Case Report

In November 2007 a 64-year old man was referred to our hospital with leucocytosis, anemia and asthenia. Comorbidities included hypertension and diabetes mellitus. At presentation, PB count showed Hemoglobin (Hb) level of 10.7 g/dl, WBC count of 42.57 × 10^9^/l (4.25 × 10^9^/l monocytes) and platelet (PLT) count of 214 × 10^9^/l. Physical examination revealed mild hepatomegaly (3 cm below costal arch), significant splenomegaly (8 cm below costal arch), no peripheral lymphoadenopathy. Abdominal ultrasound imaging confirmed increased liver size with dyshomogeneous pattern without focal lesions and markedly increase of spleen size with longitudinal diameter of 21 cm. Laboratory tests, to evaluate renal and liver functions, were normal. BM aspirate and biopsy were performed showing hypercellular bone marrow with increased number of monocytes, a variable degree of dysplasia in all three lineages, with 3% of marrow blasts. Cytogenetic analysis showed normal karyotype; JAK2V617F and bcr/abl assessment were negative. c-Kit exon 8 and 17 mutations and PDGFR alfa/beta rearrangement were also negative. TET2 mutational tests had not been performed, because still not available in our laboratory. According to these assessments, the patient was diagnosed as MPD-CMML-1.

The patient had been initially treated with Hydroxyurea, later associated with recombinant human erythropoietin for Hb decrease, obtaining a transient response. In July 2009 anemia quickly worsened (Hb 5.7 g/dl) and he became transfusion dependent. Concurrently, the patient developed a progressive leucocytosis (WBC 64.48 × 10^9^/l): BM aspirate was performed, showing an increased number of marrow blasts (11%), diagnostic for MPD-CMML-2. A combined therapy with 6-Mercaptopurine and Hydroxyurea was started, with a transient response on WBC count but not on anemia. In fact, transfusion requirement significantly increased until July 2010: at that time blood counts displayed severe anemia (Hb 6.7 g/dl), normal WBC count (3.85 × 10^9^/l, monocyte count 0,47 × 10^9^/l) with PLT count of 144 × 10^9^/l. Therefore we decided to stop ongoing cytoreductive treatment and to start the epigenetic therapy with Azacitidine after 15 days. The drug was administered subcutaneously at the approved schedule of 75 mg/m^2^/day for 7 days every 28 days (AZA 5-2-2 regimen).^10^ The patient progressively lost his transfusion dependence and obtained a partial response after 4 cycles, according to International Working Group Criteria:^12^ Hb level, WBC and PLT count were 11.1 g/dl, 10.61 × 10^9^/l and 167 × 10^9^/l, respectively. Absence of blast cells with slight degree of trilineage dysplasia was documented in BM revaluation in October 2010. Physical examination revealed a mild reduction of splenomegaly, confirmed also by the abdominal ultrasound (spleen longitudinal diameter: 18 cm). No hematologic toxicity was reported in the first four cycles.

In November 2010 the patient started the fifth Azacitidine cycle and he unexpectedly developed a severe and prolonged hematologic toxicity: on day +32 blood count displayed a serious degree of anemia (Hb 6.3 g/dl, leading to a new transfusion requirement), mild leucopenia (WBC 1.2 × 10^9^/l with absolute neutrophil count of 0.54 × 10^9^/l) and severe thrombocytopenia (PLT 7 × 10^9^/l). BM aspirate and biopsy on day + 48 excluded a leukemic transformation, showing 10% of cellularity, rare CD34+ cells and features highly suggestive of a therapy related bone marrow hypoplasia. Viral infections were also excluded: serological test for Cytomegalovirus (CMV), Ebstein-Barr Virus (EBV), Parvovirus B19, Enterovirus, Adenovirus, Herpes virus 1–2, Hepatitis B (HBV), Hepatitis C (HCV) and HIV 1–2 were negative. Transfusion support had been immediately started and granulocyte colony stimulating factor (G-CSF) was administered starting from day +49, because of lack of neutrophils recovery. Slow hematologic improvement was observed from day +62 and hematologic toxicity partially recovered from day +71; at day +90 the patient showed resolution of neutropenia (WBC 5.2 × 10^9^/l with absolute neutrophil count of 1.94 × 10^9^/l), a partial recovery of PLT count (PLT 132 × 10^9^/l) and a persistent severe transfusion dependent anaemia (Hb 7.3 g/dl). Monocyte count was 2.14 × 10^9^/l. Antibiotic and antiviral prophylaxis were administered during this prolonged cytopenia and the patient didn’t experience any infective complications. The treatment with Azacitidine was definitively stopped.

After 120 days from the last Azacitidine cycle, an increasing monocytosis (4.2 × 10^9^/l) in PB reappeared, suggesting a progression of CMML. Concomitant increase in spleen size was observed (25 cm ultrasound longitudinal diameter, with signs of secondary portal hypertension). A final attempt of reintroduction of low-dose Azacitidine (one third of the initial dose) was performed for 3 cycles, without significant hematologic improvement. The patient died in July 2011 because of gastrointestinal hemorrhage, due to an esophageal variceal rupture.

## Discussion

The efficacy of Azacitidine and Decitabine for the treatment of MDS has been confirmed by several national and international clinical trials.^7^–^9^,^12^ These studies proved efficacy of hypomethylating agents compared to best supportive care. Azacitidine significantly prolongs median time to progression to acute myeloid leukemia and improves median OS, compared to conventional care regimens.^9^–^10^ Hypomethylating agents have been used in CMML, but there are no prospective studies with sufficient number of patients with this disease.^5^,^8^

It is known from literature, that Azacitidine-related cytopenias in MDS are generally transient (median 14–16 days) and mainly reported during the first 1–2 cycles, decreasing in frequency thereafter. To those patients, who develop “early cytopenias”, it is recommended to continue Azacitidine therapy after neutrophil recovery: responder patients may develop hematologic improvement and benefit from extended treatment, until disease progression. In fact, due to the reversible effects of hypomethylating agents and to the lack of eradication of the malignant clone, the treatment should be continued until response persists.^14^,^15^ It is highly recommended to delay the therapy in case of hematologic toxicity by one or two weeks; Azacitidine dose adjustments are scheduled in some selected cases, according to blood counts observed at the beginning of the subsequent cycle. No irreversible cytopenias have been reported so far.^11^,^14^

Of note, the best treatment schedule of Azacitidine in CMML patients is still controversial, with few data regarding the incidence of adverse events, particularly concerning long term hematologic toxicity. For the first time, Costa et al.^5^ recently reported their experience concerning Azacitidine treatment in a selected group of 37 patients affected by MPD-CMML and MDS-CMML; their data confirmed encouraging activity of Azacitidine in CMML setting with an overall response rate (ORR) of 39%, showing a lower response rate and a lower OS in MPD-CMML compare to MDS-CMML patients (ORR 32% versus 55%; OS 12 months versus 23 months, respectively). The drug has been overall well tolerated with an acceptable hematologic and non-hematologic toxicity in most of the patients: 9 patients experienced some type of cytopenia, always transient. No significant differences have been shown in MDS and MPD-CMML variants, in terms of major adverse events; indeed prolonged hematologic toxicity was not reported. In this study, Costa et al.^5^ proposed a reduction of Azacitidine dosage in a subgroup of 9 patients (25%): a schedule of 100 mg/m^2^/day for 5 days (AZA 5) has been tested and resulted more convenient and equal in efficacy.^8^

In the last two years, various gene mutations were identified in CMML,^16^,^17^ although none specific for the disease. For example, the presence of TET2 mutations seems to predict a higher response rate to Azacitidine in MDS, although this does not result to a sure benefit in response duration or survival. The prognostic impact, in term of a better outcome for CMML patients whit TET2 mutations, still has to be determined and remains controversial.^17^

According to these considerations, our patient obtained a partial hematologic response to Azacitidine treatment with conventional schedule, according to IWG criteria;^12^ he gained transfusion independence without any hematologic toxicity during the first 4 cycles of therapy. Surprisingly, after cycle 5 he developed 90 days of severe and delayed bone marrow hypoplasia, conditioning drug withdrawal. The drug was subsequently used with dose reduction at the progression of disease, without any hematologic response. In our opinion, this atypical delayed and prolonged hematologic effect, is not within a predictable range of toxicity of the drug.^11^,^14^–^15^

We could suppose that previous multiple cytoreductive treatments have led to a greater sensitivity to the hypomethylation and the cytotoxic effect of the drug. In fact, many CMML-patients, unlike MDS ones, who receive Azacitidine without previous cytoreductive therapy, are usually pre-treated. Therefore, an alternative dosing schedule for CMML patients might be needed, particularly for the maintenance treatment for those patients, who achieved any hematologic response during the first 4 cycles. Maybe a reduction in the daily dose or in days of the drug infusion might be a possible solution, in order to reduce the hematologic toxicity rate.

Moreover only few reports have recently assessed the efficacy and safety of alternative dosing schedules of Azacitidine in MDS and CMML, without compromising the efficacy and the tolerability of the drug compared to the approved schedule.^5^,^8^,^18^–^19^

Lyons et al.^18^ evaluated the safety and efficacy of three alternative Azacitidine dosing schedules in MDS/CMML patients (AZA 5-2-2 at 75 mg/m2 for 7 days, AZA 5 -2 -5 at 50 mg/m2 for 10 days, AZA 5 at 75 mg/m2 for 5 days - every 4 weeks). Hematologic improvement was similar in the three arms (44% vs 45% vs 56%, in the AZA 5-2-2, AZA 5-2-5 and AZA 5, respectively). Moreover, the incidence of adverse events and hematologic toxicity was 84%, 77% and 58%, in the AZA 5-2-2, AZA 5-2-5 and AZA 5, respectively. More patients in the AZA 5 arm completed six cycles of treatment (64%) than in the AZA 5-2-2 (44%) and AZA 5-2-5 (49%). Bergua et al.^19^ analyzed the results of AZA 5 schedule in patients with high-risk MDS and acute myeloid leukemia refractory to conventional therapy. They concluded that 5 days of therapy seems as effective as 7 days in this unfavourable setting.

Therefore dose adjustments in term of daily dose or in days of the drug infusion could be proposed, for example, in pretreated CMML patients, as our case.

To our knowledge, no similar prolonged hematologic adverse events have been previously reported in literature, suggesting the need of more data, to better define the optimal timing, the best schedule of drug administration and the correct management of a prolonged therapy with Azacitidine, in CMML patients.

## References

[1] Ahmed F, Osman N, Lucas F, Neff G, Smolarek T, Bennet JM, Komrokji RS (2009). Therapy related CMML: a case report and review of the literature. Int J Hematol.

[2] Vardiman JW, Thiele J, Arber DA, Brunning RD, Borowitz MJ, Porwit A, Harris NL, Le Beau MM, Hellström-Lindberg E, Tefferi A, Bloomfield CD (2009). The 2008 revision of the World Health Organization (WHO) classification of myeloid neoplasms and acute leukemia: rationale and important changes. Blood.

[3] Bennett JM, Catovsky D, Daniel MT, Flandrin G, Galton DA, Gralnick H, Sultan C, Cox C (1994). The chronic myeloid leukaemias: guidelines for distinguishing chronic granulocytic, atypical chronic myeloid, and chronic myelomonocytic leukaemia. Proposals by the French-American-British Cooperative Leukaemia Group. Br J Haematol.

[4] Such E, Cervera J, Costa D, Solé F, Vallespí T, Luno E, Collado R, Calasanz MJ, Hernández-Rivas JM, Cigudosa JC, Nomdedeu B, Mallo M, Carbonell F, Bueno J, Ardanaz MT, Ramos F, Tormo M, Sancho-Tello R, del Canizo C, Gómez V, Marco V, Xicoy B, Bonanad S, Pedro C, Bernal T, Sanz GF (2011). Cytogenetic risk stratification in chronic myelomonocytic leukemia. Haematologica.

[5] Costa R, Abdulhaq H, Haq B, Shadduck RK, Latsko J, Zenati M, Atem FD, Rossetti JM, Sahovic EA, Lister J (2011). Activity of azacitidine in chronic myelomonocytic leukemia. Cancer.

[6] Kantarjian H, Oki Y, Garcia-Manero G, Huang X, O’Brien S, Cortes J, Faderl S, Bueso-Ramos C, Ravandi F, Estrov Z, Ferrajoli A, Wierda W, Shan J, Davis J, Giles F, Saba HI, Issa JP (2007). Results of a randomized study of 3 schedules of low-dose decitabine in higher risk myelodysplastic syndrome and chronic myelomonocytic leukemia. Blood.

[7] Fenaux P, Mufti GJ, Hellstrom-Lindberg E, Santini V, Finelli C, Giagounidis A, Schoch R, Gattermann N, Sanz G, List A, Gore SD, Seymour JF, Bennett JM, Byrd J, Backstrom J, Zimmerman L, McKenzie D, Beach C, Silverman LR, International Vidaza High-Risk MDS Survival Study Group (2009). Efficacy of azacitidine compared with that of conventional care regimens in the treatment of higher-risk myelodysplastic syndromes: a randomised, open-label, phase III study. Lancet Oncol.

[8] Greco M, Criscuolo M, Fianchi L, Fabiani E, Pagano L, Voso MT (2011). 5-azacytidine in Chronic Myelomonocytic Leukemia: Case report and review of literature. Mediterr J Hematol Infect Dis.

[9] Fenaux P, Bowen D, Gatterman N, Hellström-Lindberg E, Hofmann WK, Pfeilstöcker M, Sanz G, Santini V (2010). Practical use of azacitidine in higher-risk myelodysplastic syndromes: An expert panel opinion. Leuk Res.

[10] van der Helm LH, Alhan C, Wijermans PW, van Marwijk Kooy M, Schaafsma R, Biemond BJ, Beeker A, Hoogendoorn M, van Rees BP, de Weerdt O, Wegman J, Libourel WJ, Luykx-de Bakker SA, Minnema MC, Brouwer RE, Croon-de Boer F, Eefting M, Jie KS, van de Loosdrecht AA, Koedam J, Veeger NJ, Vellenga E, Huls G (2011). Platelet doubling after the first azacitidine cycle is a promising predictor for response in myelodysplastic syndromes (MDS), chronic myelomonocytic leukaemia (CMML) and acute myeloid leukaemia (AML) patients in the Dutch azacitidine compassionate named patient programme. Br J Haematol.

[11] Santini V, Fenaux P, Mufti GJ, Hellström-Lindberg E, Silverman LR, List A, Gore SD, Seymour JF, Backstrom J, Beach CL (2010). Management and supportive care measures for adverse events in patients with myelodysplastic syndromes treated with azacitidine. Eur J Haematol.

[12] Cheson BD, Bennett JM, Kantarjian H, Pinto A, Schiffer CA, Nimer SD, Löwenberg B, Beran M, de Witte TM, Stone RM, Mittelman M, Sanz GF, Wijermans PW, Gore S, Greenberg PL, World Health Organization (WHO) international working group (2000). Report of an international working group to standardize response criteria for myelodysplastic syndromes. Blood.

[13] Kantarjian H, Issa JP, Rosenfeld CS, Bennett JM, Albitar M, DiPersio J, Klimek V, Slack J, de Castro C, Ravandi F, Helmer R, Shen L, Nimer SD, Leavitt R, Raza A, Saba H (2006). Decitabine improves patient outcomes in myelodysplastic syndromes: results of a phase III randomized study. Cancer.

[14] Santini V, Alessandrino PE, Angelucci E, Barosi G, Billio A, Di Maio M, Finelli C, Locatelli F, Marchetti M, Morra E, Musto P, Visani G, Tura S, Italian Society of Hematology (2010). Clinical management of myelodysplastic syndromes: update of SIE, SIES, GITMO practice guidelines. Leuk Res.

[15] Silverman LR, Fenaux P, Mufti GJ, Santini V, Hellström-Lindberg E, Gattermann N, Sanz G, List AF, Gore SD, Seymour JF (2011). Continued azacitidine therapy beyond time of first response improves quality of response in patients with higher-risk myelodisplastic syndromes. Cancer.

[16] Bacher U, Haferlach T, Schnittger S, Kreipe H, Kröger N (2011). Recent advances in diagnosis, molecular pathology and therapy of chronic myelomonocytic leukaemia. Br J Haematol.

[17] Braun T, Itzykson R, Renneville A, de Renzis B, Dreyfus F, Laribi K, Bouabdallah K, Vey N, Toma A, Recher C, Royer B, Joly B, Vekhoff A, Lafon I, Sanhes L, Meurice G, Oréar C, Preudhomme C, Gardin C, Ades L, Fontenay M, Fenaux P, Droin N, Solary E, Groupe Francophone des Myélodysplasies (2011). Molecular predictors of response to decitabine in advanced chronic myelomonocytic leukemia: a phase 2 trial. Blood.

[18] Lyons RM, Cosgriff TM, Modi SS, Gersh RH, Hainsworth JD, Cohn AL, McIntyre HJ, Fernando IJ, Backstrom JT, Beach CL (2009). Hematologic response to three alternative dosing schedules of azacitidine in patients with myelodysplastic syndromes. J Clin Oncol.

[19] Bergua J, Arcos Carmona MJ, Prieto Fernandez J, Cabrera Silva C, Carnicero F, Fernandez-Leyva H, Bengochea Miranda ML, Martin-Mateos ML, Izquierdo F, Gil Esparraga E, Bermejo Vega N, Garcia Blanco MJ (2011). Azacytidine 75 mg/m2 × 5 day in high-risk myelodisplastic syndrome and acute myeloid leukemia refractory/relapsed patients: Results from a single centre. Leukemia Research.

